# Polyphenols in Exercise Performance and Prevention of Exercise-Induced Muscle Damage

**DOI:** 10.1155/2013/825928

**Published:** 2013-07-24

**Authors:** Marco Malaguti, Cristina Angeloni, Silvana Hrelia

**Affiliations:** Department for Life Quality Studies—Alma Mater Studiorum—University of Bologna, Via Irnerio 48, 40126 Bologna, Italy

## Abstract

Although moderate physical exercise is considered an essential component of a healthy lifestyle that leads the organism to adapt itself to different stresses, exercise, especially when exhaustive, is also known to induce oxidative stress, inflammation, and muscle damage. Many efforts have been carried out to identify dietary strategies or micronutrients able to prevent or at least attenuate the exercise-induced muscle damage and stress. Unfortunately most studies have failed to show protection, and at the present time data supporting the protective effect of micronutrients, as antioxidant vitamins, are weak and trivial. This review focuses on those polyphenols, present in the plant kingdom, that have been recently suggested to exert some positive effects on exercise-induced muscle damage and oxidative stress. In the last decade flavonoids as quercetin, catechins, and other polyphenols as resveratrol have caught the scientists attention. However, at the present time drawing a clear and definitive conclusion seems to be untimely.

## 1. Introduction

Physical exercise, as well as nutritional behavior, is now widely considered to be an essential component of a healthy lifestyle. Moreover moderate exercise and an active lifestyle have been demonstrated to be useful in the primary and secondary prevention of cardiovascular diseases [1], type II diabetes [2], metabolic syndrome [3], and neurodegenerative diseases like Alzheimer's disease [4, 5]. It is now well known that regular and moderate exercise represents a mild source of stress able to induce an adaptive response. More importantly it seems that this adaptive response provides protection against other stressors [6], explaining how the practice of regular and moderate exercise plays a key role in the prevention of chronic and degenerative diseases.

The organism ability to adapt itself to stress is known as *hormesis*. This term refers to a J-shaped or inverted U-shaped dose-response curve produced by biological systems exposed to a stressor. Radak et al. [7] have firstly extended the *hormesis* theory to the exercise-induced effects. According to this theory, adaptation occurs only when the stressor dose, exercise bout, is within a specific range and followed by rest period. When the stressor is absent no adaptation can occur. On the other hand when exercise bouts are too heavy or not followed by rest periods (overtraining), pathological conditions as muscle damage, oxidative stress, and inflammation can occur as well. For a comprehensive review on this topic please refer to Radak et al. [8].

After many years of intensive research it is now well documented that exercise induces reactive oxygen species (ROS) production resulting in oxidative stress as clearly demonstrated by the induction of lipid peroxidation [9–12], superoxide anion generation through xanthine oxidase activation, and the increase in oxidized/reduced glutathione (GSSG/GSH) ratio [13, 14].

Many authors have investigated which metabolic pathways are influenced by exercise and if exercise may induce an adaptive response able to prevent, or at least delay, the onset of degenerative diseases. The potentially harmful condition of imbalance of the redox homeostasis plays a fundamental role in the organism adaptive response to exercise. In 2008, for the first time, Gomez-Cabrera et al. [15] defined moderate exercise as an antioxidant, explaining that the mild burst of ROS, generated by training, acts as a signal responsible for the activation of signaling pathways that lead to the induction of antioxidant enzymes in human tissue. Other studies have investigated the pathways involved in this process, and it is now well documented that the major players are nuclear factor kB (NF-kB), the phosphoinositide 3-kinase/Akt (PI3 K/Akt), p53, heat shock proteins (HSPs), and mitogen-activated protein kinases (MAPKs) [16–19].

If it is commonly accepted that moderate exercise and training are key components of a healthy lifestyle and help to prevent or delay the onset of pathological conditions, it is now clear that these beneficial effects are lost when the exercise becomes exhaustive, indicating that the exercise intensity and duration are responsible for the beneficial or detrimental effects of physical activity.

A great body of literature has demonstrated that exhaustive exercise causes oxidative stress, inflammatory response, and structural damage to muscle cells, evidenced by an increase in the plasma activity of cytosolic enzymes, namely, lactic dehydrogenase (LDH) and creatine kinase (CK) [11, 20, 21]. So, many studies have investigated the possibility to prevent the exercise-induced oxidative stress and muscle damage through nutritional intervention, mainly using antioxidant vitamins and polyunsaturated fatty acids [22–29].

The effects of both Vitamins C and E have been investigated in a wide range of exercise conditions, using a variety of supplementation strategies, timing, and dosage. Even though antioxidant vitamin supplementation seems to be a reasonable strategy to reduce or prevent tissues damage in active muscles, there are only few and weak results able to support this thesis [22].

Thompson et al. [24] demonstrated that an acute supplementation (1000 mg) of Vitamin C 2 h prior to a 90 min intermittent shuttle running test does not affect the increases in serum CK level. Despite that some authors [23] found an increase in plasma total antioxidant capacity in subjects treated with 1000 mg Vitamin C for 2 weeks prior to 2.5 h cycling at 60% VO_2_max⁡, others [30] found a reduction in IL-6 and malondialdehyde (MDA) plasma levels in subjects treated with 400 mg Vitamin C for 2 weeks prior to a 90 min intermittent shuttle running test. Similarly to Vitamin C, the effects of Vitamin E on exercise-induced muscles damage are still under debate, and the results of different studies are not in agreement. Cannon et al. [26, 31] found that the supplementation of 800 IU of *α*-tocopherol for 48 days does not prevent plasma CK release due to 45 min downhill running in young men, even though it reduces the secretion of IL-1*β* and IL-6. Two studies reported a positive effect of Vitamin E on exercise-induced muscles damage; Beaton et al. [25] and McBride et al. [32] found, respectively, that 30 days and 14 days supplementation of 1200 IU *α*-tocopherol reduces CK level in serum and plasma after different exercises. These results disagree with those published by Avery et al. [33] which found that 1200 IU *α*-tocopherol, 21 days before and 10 days after exercise, increases CK serum level when subjects undergo exercise bouts.

When Vitamins C and E are supplemented in combination some positive effect can be obtained, but, as recently reviewed [22], for each study providing positive effects [34] there are other studies that provide no effect [35, 36].

Only recently the attention has been shifted to the effects of nutraceutical bioactive compounds as polyphenols. Polyphenols are a class of organic chemical compounds, mainly found in plants, characterized by the presence of multiples of phenol structural units. Recently a great body of literature has underlined a potential relationship between bioactive compounds from plant foods and the prevention of cardiovascular and neurodegenerative diseases and other pathological conditions [37–41].

This review will summarize some of the actual knowledge on polyphenolic compounds that have been demonstrated both to exert a significant effect in exercise-induced muscle damage and to play a biological/physiological role in improving physical performance.

## 2. Flavonoids

Among nutraceutical compounds, flavonoids are the mainly studied ones for their positive effects on human health. Some of them have been proposed to be beneficial in exercise and exercise performance. Flavonoids are a family of plant bioactive compounds that share a common backbone. The flavonoid family includes many different subclasses: flavones, flavonols, flavanones, flavanones, isoflavones, and anthocyanidins; in Table 1 some examples are reported for each subclass.

On the left column flavonoid subclasses are reported; on the right some examples for each subclass are listed.

### 2.1. Flavonols

**Figure 1:**
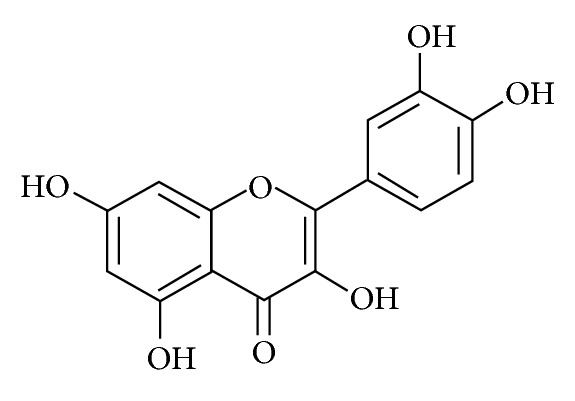
Quercetin structure. Quercetin (3,5,7,3′,4′-pentahydroxyflavone) is a typical flavonol-type flavonoid and forms the backbone for many other flavonoids. Flavonoids are characterized by 2 benzene rings connected by an oxygen-containing pyrene ring.

Flavonols are present in human nutrition as both glycosides and aglycone forms, and it has been estimated that the daily intake is within the range of 20–50 mg/die in Western population. Of these flavonols quercetin (Figure 1) accounts for about 13.82 mg/die [42], resulting in being one of the most abundant flavonols in Western diet.

Quercetin, mainly present as quercetin glycosides (rutin, spiraeoside, troxerutin quercitrin, isoquercitin, and hyperoside), is widely distributed in plant food; it is found in apples, berries, onions, grapes, tea, and tomatoes as well as in some medicinal plants as *Hypericum perforatum* and *Gingko biloba* [43–46].

Recent studies suggest that quercetin bioavailability is much higher than that originally thought [47]. Quercetin, assumed both as purified dietary supplement or as natural food source, has been clearly demonstrated to increase its plasma levels in humans, even though a high interindividual variability in plasma quercetin response has been reported [48–52]. Quercetin aglycone is a lipophilic molecule able to diffuse through the enterocyte membranes. Quercetin glucosides are easily hydrolyzed both in the mouth, during chewing, and in the gut, thanks to beta-glucosidase enzymes [53]. The overall result is that quercetin absorbed as aglycone is much higher than that expected from its food content [47].

Quercetin and other flavonoids have been reported to exert a variety of biological activities often related to their antioxidant nature. McAnulty et al. [54] have investigated the effects of quercetin supplementation in cycling athletes. Subjects were supplemented with 1000 mg/die quercetin or placebo for 6 weeks before and during 3 days in which they cycle for 3 h/die. In blood and plasma, F2-isoprostanes, nitrite, ferric-reducing ability, trolox equivalent antioxidant capacity, and C-reactive protein were analyzed before and after exercise. The authors concluded that cycling induces a strong increase in blood biomarkers of oxidative stress and inflammation, but, despite previous data demonstrating potent antioxidant actions of quercetin in *in vitro* and animal models [55–59], long-term quercetin supplementation was not able to exert any preventive effect on exercise-induced oxidative stress and inflammation biomarkers.

Similar conclusions have been reached when the effects of quercetin in association with catechin, isoquercetin, and PUFA were evaluated in an acute crossover study involving 20 endurance athletes supplemented with an oral dose providing 1000 mg quercetin [60]. Athletes were supplemented 15 min prior to a 2-hour run. Authors analyzed before, immediately after, and one hour after exercise plasma quercetin level, C-reactive protein, IL-6, and other cytokines and inflammatory biomarkers, confirming a good quercetin bioavailability but supporting that it does not prevent postexercise inflammation.

In another study [61] the effects of quercetin on inflammation and exercise-induced muscle damage in 39 trained cyclists were evaluated. In this study quercetin was provided in association with Vitamin C, niacinamide, and folic acid (Q) or in association with Vitamin C, niacinamide, folic acid, catechin, isoquercetin, and PUFA (Q-EGCG) for 2 weeks before, during, and 1 week after a 3 days period in which the subject cycled for 3 h/die. Q-EGCG association was specifically designed to improve quercetin bioavailability and extend its bioactive effects. Results confirm those previously published [54], showing that a long-term quercetin (Q) supplementation is not able to modulate serum C-reactive protein level and plasma IL-6 concentration and prevent CK relies. The Q-EGCG association decreased almost all biomarkers but not CK levels.

Recently Askari et al. [62] published a double-blind clinical trial on 60 male students with an athletic history of at least 3 years. They found that 500 mg quercetin plus 250 mg Vitamin C daily treatment lasting for 8 weeks was able to improve some markers such as lean body mass, basal metabolic rate, and total energy expenditure. In a previous study the same authors showed that quercetin plus Vitamin C treatment reduced CK plasma levels after treadmill exercise [63].

Beside the possibility to partially prevent exercise-induced muscle damage and inflammation, in the last decade a considerable effort has been performed to analyze the possibility that quercetin supplementation could improve aerobic exercise performance in human.

The rationale behind this hypothesis is given by the knowledge that some polyphenols as catechins, resveratrol, quercetin, and curcumin have been shown to activate sirtuins (SIRT1). SIRT1 activation modulates a variety of biological and physiological processes including skeletal muscle function and mitochondria biogenesis [64, 65].

Early human and animal studies reported a correlation between quercetin supplementation, endurance capacity, and mitochondrial biogenesis improvement. Davis et al. [66] published promising results showing that 7 days quercetin treatment (12.5 or 25 mg/kg b.w.) increases the expression of genes associated with mitochondrial biogenesis (PGC-1a and SIRT1), mitochondrial DNA content, and cytochrome-C concentration, both at muscle and brain levels in mice. Beside these biological data, quercetin-treated mice showed a significantly higher time to fatigue, in a treadmill running test, than their placebo-treated counterparts. A more recent study has evaluated quercetin ability to increase endurance capacity and VO_2_max⁡ in a cross-over protocol on healthy but untrained volunteers [67]. Data indicate that a 7 days 1000 mg/die quercetin supplementation is responsible for an improvement (13.2%) in time to fatigue, during a cycling test, and for a 3.9% increase in VO_2_max⁡. Authors hypothesized a quercetin-induced increase in mitochondrial biogenesis that would lead to an increase in endurance capacity through a shift toward fat oxidation during exercise, but they did not investigate any mitochondrial biogenesis biomarkers.

Other studies have analyzed the effect of quercetin on exercise performance, some reporting positive effects [68, 69], while others do not [70–73], but to our knowledge an increase in mitochondrial biogenesis has not been reported in human even though Neiman et al. [68] have shown a modest and insignificant increase in relative mitochondrial DNA copy number following quercetin supplementation.

In a recent meta-analysis Kressler et al. [74] have demonstrated that quercetin can improve endurance capacity in humans, but they concluded that the benefit magnitude is only trivial to small. Because the “mitochondrial biogenesis” theory that could explain the quercetin-induced endurance capacity has not been demonstrated, some other mechanisms have been suggested. According to its ability to bind and act as antagonist at adenosine receptor level, quercetin may improve exercise performance in a caffeine-like manner [75, 76]. However, up to now, this hypothesis has still to be demonstrated, and the only study that has compared caffeine versus quercetin for their ability to improve exercise performance in the heat has failed to find any significant result [77].

### 2.2. Catechins

Although quercetin is the most studied flavonoid in relation to exercise, other molecules are under investigation for their ability to prevent exercise-induced muscle damage and to affect physical performance. Among them catechins have shown to exert some effects at least in animal models.

**Figure 2:**
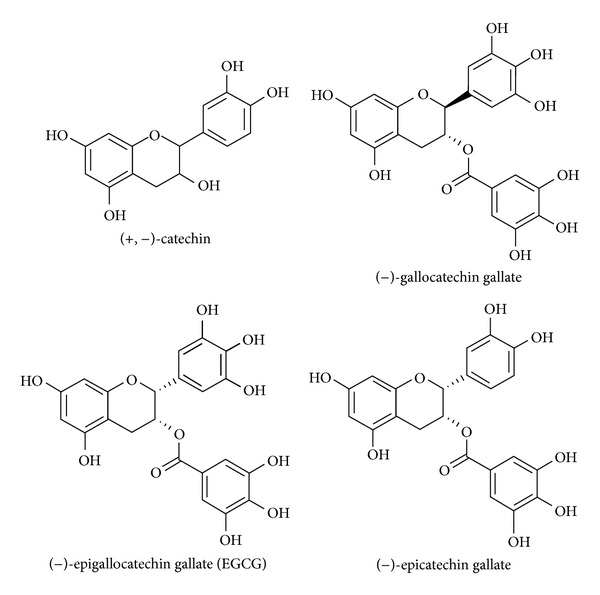
Chemical structure of the main catechins. Catechins are characterized by two benzene rings and a dihydropyran heterocycle with a hydroxyl group in position 3. Thanks to the presence of this hydroxyl group catechins are also called flavan-3-ol. Catechin gallates are gallic acid esters of the catechins as epigallocatechin gallate and gallocatechin gallate, which are typically found in tea.

As all other flavonoids, catechins represent a family of plant secondary metabolites and belong to the flavanols subclass. Catechins (Figure 2) are easily found in edible foods and plants such as green tea and cacao. Catechin family consists mainly of epigallocatechin gallate (EGCG), gallocatechin gallate, and epicatechin gallate and have been demonstrated to be bioavailable and to possess various biological properties such as cardioprotective [78, 79], antiatherogenic [80], and anticarcinogenic effects [81, 82].

Some evidence on catechins ability to modulate exercise-induced muscle damage is rising. Haramizu et al. [83] have recently shown that 8 weeks catechins treatment is able to attenuate loss of muscle force, exercise-induced muscle damage, and oxidative stress biomarkers (CK, LDH, MDA) and to significantly sustain GSH/GSSG ratio after downhill running exercise in senescence-accelerated mice.

Catechins ability to modulate the exercise performance, both in animals and humans, has also been investigated. Murase et al. [84] have shown that green tea extract improves, in a dose-dependent manner, the time to exhaustion in BALB/c mice undergoing a swimming test after 7 weeks treatment. They have also demonstrated, through indirect calorimetry and biochemical analysis, that green tea extract induces a more efficient use of lipids as suggested by the increase in oxygen consumption, *β*-oxidation activity in muscle, and fat oxidation, suggesting that the green tea extract-induced lipid oxidation and mobilization are responsible for the increase in endurance capacity. These findings have been deepened, and recently it has been observed that EGCG treatment increases the expression of genes involved in mitochondrial fat oxidation at muscle level in high-fat fed mice [85]. Although most studies on green tea have been performed in animals, a considerable amount of data are now available in humans. Dulloo et al. [86] showed that a green tea extract rich in catechins and caffeine increases the daily energy expenditure in humans. More recently, an acute dose of green tea extract has been evaluated on healthy untrained men in a 30 min cycling test at 60% VO_2_max⁡ [87]. Results demonstrated that green tea extract improves fat oxidation and insulin sensitivity during moderate exercise. Other recent findings report that short-term EGCG supplementation increases VO_2_max⁡ in adult humans [88], while Dean et al. [89] concluded that 6 days EGCG treatment does not significantly affect fat oxidation during a 60 min cycling exercise at 60% VO_2_max⁡ in moderately well-trained men. In a randomized, double-blind crossover study, 10 endurance-trained subjects exercised for 2 hours at 50% of their maximal power output before and after 3 weeks of green tea extract supplementation [90]. The treatment did not influence fat and energy metabolism biomarkers (oxygen uptake, respiratory exchange ratio, and energy expenditure), cytokines and inflammatory parameters (IL-6, C-reactive protein), and oxidative stress biomarkers (thiobarbituric acid-reactive substances, oxidized LDL). However,plasma CK level was significantly reduced. Recently Jowko at al. [91] published data obtained treating a group of 16 soccer players with a single dose of 640 mg green tea catechins. Athletes involved in the study performed a muscle-endurance test consisting in 3 bouts to exhaustion of bench press and back squat. Prior to and after exercise test plasma levels of thiobarbituric acid-reacting substances, uric acid, total catechins, total antioxidant status, and CK activity were analyzed.

None of the analyzed biomarkers was affected by the ingestion of green tea catechins, suggesting that the 640 mg dose was too low to attenuate exercise-induced oxidative stress and muscle damage. Green tea catechins-treated players were able to perform a higher number of lift repetitions during the test.

Taken together, data from available studies seem to suggest that catechins can improve physical performance particularly in term of endurance capacity and VO_2_max⁡ in untrained subjects, but the same results cannot be reached in physically active people and well-trained athletes.

## 3. Other Polyphenols

**Figure 3:**
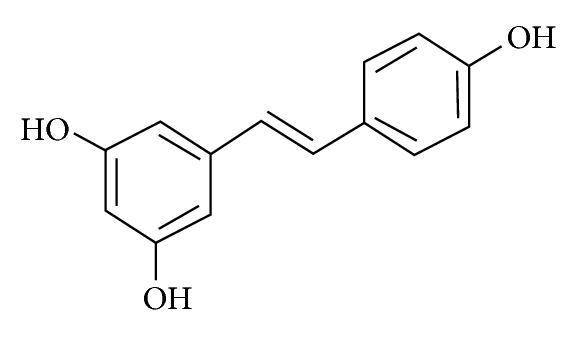
Resveratrol structure. Resveratrol (3,5,4′-trihydroxystilbene) belongs to the stilbene group. It exists both as *cis-* and *trans-*isomers.

Resveratrol is a well-known bioactive compound able to induce a wide variety of biological responses. It has shown beneficial effects against most degenerative and cardiovascular diseases from atherosclerosis, hypertension, ischemia/reperfusion, heart failure, diabetes, obesity, aging and neurodegenerative diseases [40, 92]. Resveratrol, *3,5,4*′*-trihydroxystilbene* (Figure 3), is a natural phenol present in grape skin and seeds and in grape-derived products like red wine. 

Only few studies have investigated resveratrol ability to modulate exercise performance and some evidence suggests that it could play a role improving endurance capacity. It has been demonstrated that after 12 weeks treatment resveratrol prevents the decline in running time to exhaustion, in oxygen consumption, and in lipid oxidation in a senescence-accelerated mice model (SAMP1). Data shown in this study demonstrate that the resveratrol induction of mitochondrial biogenesis (suggested by the mRNA levels of genes involved in mitochondrial biogenesis and energy metabolism, namely, peroxisome proliferator-activated receptor coactivator-1, cytochrome-C, and medium-chain acyl-CoA dehydrogenase) is responsible for the prevention of ageing-related performance impairment [93]. Accordingly, Dal-Ros et al. [94] have recently demonstrated that chronic red wine polyphenols intake prevents aging-induced performance decline in rats.

**Figure 4:**
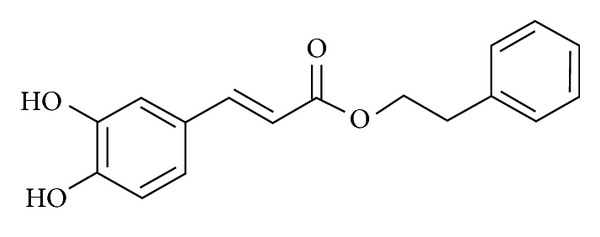
Caffeic acid phenethyl ester (CAPE) structure. CAPE is a polyphenolic compound primarily found in propolis.

Recently some authors have focused their interest on the effect of caffeic acid, especially in its phenethyl ester form (CAPE) (Figure 4). Shen et al. [95] demonstrated that 10 mg/kg b.w. CAPE oral treatment protects rat muscle tissues from exercise-induced damages. In this study the protocol was represented by an intermittent downhill eccentric exercise able to induce both muscle damages and inflammation as demonstrated by the increase in CK serum level, NF-kB activation, iNOS and COX-2 expression, and IL-*β* level. Moreover, an *ex vivo* study by Chen et al. [96] shows that CAPE exhibits protective effect against hyperthermal stress, known to impair endurance capacity, in isolated peripheral blood mononuclear cells from competitive cyclists. Pretreatment of mononuclear cells with CAPE reduced hyperthermia-induced necrosis, superoxide production, glutathione depletion, and intracellular superoxide anion production in a dose-dependent manner.

Beside studies on single polyphenols, some researches have been focused on the effects of polyphenol mixtures obtained from fruits, plants, or algae. Hurst et al. [97] have investigated the effects of a blueberry extract on skeletal muscle cultured cells showing a dose-dependent protective effect on oxidative stress. Furthermore, Nakazato et al. [98] found that rats fed an apple polyphenol-enriched diet for 3 weeks showed a better oxidative stress biomarkers profile (thiobarbituric acid reactive substances and protein carbonyls), a lower force deficit, and earlier recovery after strenuous lengthening muscle contractions with respect to their control counterparts. Promising results have been published by Swamy et al. [99] on the effect of pomegranate peel extract supplementation in rats. In this study rats were supplemented for 28 days with 25 mg/die pomegranate peel extract, rich in polyphenols, and were forced to swim until exhaustion. Interestingly, they recorded a strong increase in time to exhaustion among rats fed pomegranate extract with respect to their control counterparts. Authors also found that pomegranate extract fed rats had a higher glycogen and ATP muscular content with respect to controls, and they hypothesized that pomegranate polyphenols are responsible for a better glucose utilization resulting in a longer swimming time to exhaustion. Moreover they found that LDH and CPK serum levels were reduced in pomegranate extract fed rats, suggesting a protective role of pomegranate polyphenols against exhaustive exercise-induced muscle damage.

Dark-chocolate polyphenols have been ascribed to be responsible for some positive effects of dark-chocolate consumption during exercise. A study investigated the effects of dark-chocolate (80 g/die for 2 weeks) consumption on oxidative stress biomarkers after prolonged exhaustive exercise. Plasma F2-isoprostanes were significantly lower both at exhaustion and after 1 hour recovery in dark-chocolate-supplemented subjects with respect to their control counterparts. Moreover dark-chocolate consumption was associated with lower oxidized low-density lipoprotein levels before and after exercise and with an increase in free fatty acids levels during exercise, even though the time to exhaustion was not significantly affected [100]. Similar conclusions were drawn when Davison et al. [101] investigated the acute effects of preexercise dark-chocolate consumption. In this study dark chocolate prevents the exercise-induced increase in plasma F2-isoprostane. These results suggest that dark-chocolate intake reduces exercise-induced oxidative stress biomarkers.

Recently it has been found that *Ecklonia cava *(a species of brown alga present in the ocean of Japan and Korea) polyphenols acute preexercise supplementation induces a slight but significant increase in time to exhaustion in healthy human subjects [102].

## 4. Conclusion

Overall, the available literature seems to suggest that nutraceutical bioactive compounds as polyphenols, known for their effects on degenerative and chronic diseases, can also provide protection against exercise-induced muscle damage and oxidative stress thanks to their antioxidant and anti-inflammatory properties. However the possibility to improve the exercise performances remains unclear. Even though this topic is extremely fascinating and has attracted a great research effort scientific data do not allow to draw a clear conclusion. On one hand, *in vitro* and *in vivo* animal studies suggest that polyphenols could really play a role in improving endurance performance; on the other hand, most human trials do not support this hypothesis and have failed to demonstrate that nutraceuticals as quercetin, catechins, or resveratrol can really affect exercise performance and VO_2_max⁡. The reason for this discrepancy has still to be fully elucidated; however, some hypotheses are possible. Human studies often differ from each other in the training level of enrolled subjects; some studies have been carried on well-trained athletes while others on healthy but untrained subjects. Among recent studies those involving untrained subjects seem to have obtained better results on improving endurance capacity, although Kressler et al. [74], in their meta-analysis on quercetin, suggest that the variability among studies does not appear to be associated with the initial subject fitness level. Moreover while quercetin and catechins effects have been investigated both in animal and in human studies other polyphenols need to be further studied in human trials to reach a clear and unambiguous conclusion. 

## Figures and Tables

**Table 1:** Flavonoid classification.

Flavonoid subclasses	Representative compounds
*Flavonols *	*Quercetin, Kaempferol *
*Flavones *	*Apigenin, Luteolin *
*Flavanols *	*Epicatechin, Gallocatechin *
*Flavanones *	*Naringenin, Hesperidin *
*Isoflavones *	*Daidzein, Genistein *
*Anthocyanidins *	*Cyanidin *
